# Autophagy Modulators in Cancer Therapy

**DOI:** 10.3390/ijms22115804

**Published:** 2021-05-28

**Authors:** Kamila Buzun, Agnieszka Gornowicz, Roman Lesyk, Krzysztof Bielawski, Anna Bielawska

**Affiliations:** 1 Department of Biotechnology, Faculty of Pharmacy, Medical University of Bialystok, 15-089 Bialystok, Poland; kamila.buzun@umb.edu.pl (K.B.); anna.bielawska@umb.edu.pl (A.B.); 2 Department of Public Health, Dietetics and Lifestyle Disorders, Faculty of Medicine, University of Information Technology and Management in Rzeszow, 35-225 Rzeszow, Poland; dr_r_lesyk@org.lviv.net; 3 Department of Synthesis and Technology of Drugs, Faculty of Pharmacy, Medical University of Bialystok, 15-089 Bialystok, Poland; kbiel@umb.edu.pl

**Keywords:** autophagy, autophagy inhibitors, autophagy activators, cancer, cancer therapy

## Abstract

Autophagy is a process of self-degradation that plays an important role in removing damaged proteins, organelles or cellular fragments from the cell. Under stressful conditions such as hypoxia, nutrient deficiency or chemotherapy, this process can also become the strategy for cell survival. Autophagy can be nonselective or selective in removing specific organelles, ribosomes, and protein aggregates, although the complete mechanisms that regulate aspects of selective autophagy are not fully understood. This review summarizes the most recent research into understanding the different types and mechanisms of autophagy. The relationship between apoptosis and autophagy on the level of molecular regulation of the expression of selected proteins such as p53, Bcl-2/Beclin 1, p62, Atg proteins, and caspases was discussed. Intensive studies have revealed a whole range of novel compounds with an anticancer activity that inhibit or activate regulatory pathways involved in autophagy. We focused on the presentation of compounds strongly affecting the autophagy process, with particular emphasis on those that are undergoing clinical and preclinical cancer research. Moreover, the target points, adverse effects and therapeutic schemes of autophagy inhibitors and activators are presented.

## 1. Introduction

Autophagy, directly translated as ‘self-eating’, is an evolutionary conservative process, found in all eukaryotic cells—from single-cell yeasts to much more complex multicellular mammalian organisms [1]. The introduction of the term ‘autophagy’ was proposed in February 1963 during the conference titled ‘Ciba Foundation Symposium on Lysosomes’ which took place in London [2]. This process participates in intracellular degradation of damaged or redundant proteins with a long half-life as well as other unnecessary cytoplasm components [3,4]. Autophagy provides an organism’s homeostasis and prevent it from redundant components accumulation inside the cell [5].

Moreover, this process is involved in surfactant formation or red blood cells ripening [3]. Following the Nomenclature Committee on Cell Death, in 2018 the term ‘autophagy-dependent cell death (ADCD)’ was introduced. ADCD is a type of regulated cell death in which functional autophagic markers such as increased degradation of autophagosomal substrates or LC3 (Light Chain protein 3) lipidization occurs [6]. Interestingly, unlike necrosis or apoptosis, autophagy-dependent cell death is not synonymous exclusively with cell death. Under stressful condition such as hypoxia, nutrient deficiency or chemotherapy, this process can become the strategy for cell survival [5]. ADCD occurs in all eukaryotic cells performing important functions, for example, it is an adaptation mechanism to stressful conditions, as it provides cells with a constant supply of nutrients essential for sustaining key life processes. Additionally, through the elimination of redundant cytoplasm components and the adjustment of the endoplasmic reticulum size, ADCD participates in maintaining the intracellular homeostasis. Furthermore, ADCD is involved in tissue-specific processes, such as erythrocyte ripening or intracellular surfactant formation [3] and also protects the organism from viruses or bacteria multiplication [7,8].

Autophagy-dependent cell death, through its selective and non-selective mechanisms of degradation of pathogens, organelles and various biomolecules (nucleic acids, lipids, carbohydrates and proteins) constitutes the main catabolic system of eukaryotic cells [9,10]. As one of the key elements in maintaining cell homeostasis and health, this process also plays an important role in tumor suppression or genome integrity [11].

The first part of the following paper will provide a brief description of the different types of autophagy. Thereafter, we will focus primarily on the classification and characterization of compounds whose molecular target is autophagy—those undergoing clinical and preclinical trials. Moreover, the target points, adverse effects and therapeutic schemes of autophagy inhibitors and activators are presented in tables.

## 2. Types of Autophagy

Based on the differences in the mechanism of delivery of redundant cytoplasm components to lysosomes, four basic types of autophagy can be distinguished: macroautophagy, selective autophagy, microautophagy and chaperone-mediated autophagy (CMA). The term ‘autophagy’, commonly used in various papers, refers to macroautophagy—this will apply to the following publication as well.

### 2.1. Macroautophagy

**Figure 1:**
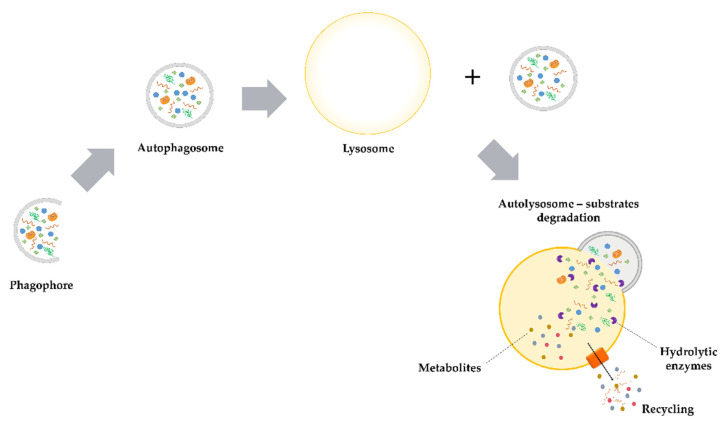
Macroautophagy. The C-shaped expansion of the double membrane results in the formation of an autophagosome. This small ‘vesicle’ contains redundant or damaged organelles, cellular fragments or proteins. In the next step, the formed autophagosome fuses with the lysosome and an autolysosome is formed. Inside the created structure, all components are degraded by hydrolytic enzymes.

Macroautophagy is the most widespread type of autophagy which is controlled by autophagy-related (*ATG*) genes. The first ATG genes were identified in yeast. Interestingly, 14 from 32 of described Atg yeast proteins are homologous to those proteins found in mammals [12,13]. This process is regulated by several pathways sensitive to the presence or deficiency of nutrients. Substances such as insulin, amino acids or AMPK (5′ adenosine monophosphate-activated protein kinase) act through the protein serine-threonine kinase mTOR (mammalian target of rapamycin). When the natural cellular environment is rich in essential nutrients, the regulatory mTOR pathway is activated, which in turn leads to the inhibition of autophagy and stimulates cells to proliferation [14]. *ATG* genes are responsible for the regulation of the autophagosomes production [15]. During the autophagy process (Figure 1), the cytoplasm fragment is surrounded by a forming C-shaped double membrane. Both ends of the membrane (known as phagophore) extend and close inside the fragment of cytoplasm with whole organelles or proteins with a long half-life. This results in the formation of 300–900 nm bubble (autophagosome), which then undergoes a maturation process. During the maturation autophagosomes and lysosomes merge to form autolysosomes (autophagolysosomes). Inside the autolysosomes, using hydrolytic lysosomal enzymes, the degradation of the macromolecular substrates to fatty acids and amino acids occurs [16,17].

Macroautophagy may play a dual role in tumorigenesis. Depending on the tumor type, its genetic background, developmental stage or tumor microenvironment, it can inhibit or stimulate tumor cell growth. Elimination of damaged organelles, aggregated or unformed proteins and oncogenic proteins prevent tumor initiation and therefore constitutes a tumor suppressor effect of autophagy. On the other hand, autophagy may also promote tumorigenesis through, for example, cytoprotective effects in response to used chemotherapeutics or metabolite recycling that promotes tumorigenesis, proliferation, or tumor metastasis [18]. With the identification of *Beclin 1*, a key gene involved in the autophagy process, it became possible to discover the connection between autophagy and various human cancers. Monoallelic deletion of *Beclin 1*, a tumor suppressor, is observed in hepatocellular carcinoma, ovarian and breast cancer [19,20,21,22]. Reduced expression of *Beclin 1* in tumor tissues was observed in 44 patients with hepatocellular carcinoma. Based on these observations, it was concluded that autophagy may lead to inhibition of tumorigenesis [22].

### 2.2. Selective Autophagy

**Figure 2:**
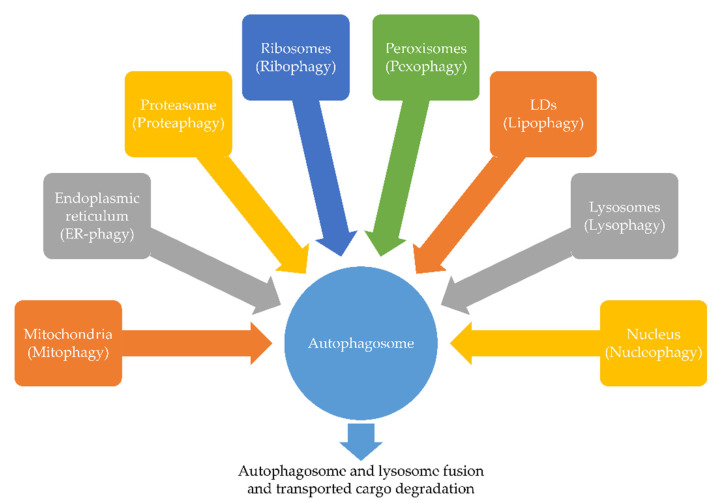
Selective forms of autophagy. Depending on the degraded organelle we can distinguish: mitophagy, ER-phagy, proteaphagy, ribophagy, pexophagy, lipophagy, lysophagy and nucleophagy.

As mentioned in the introduction, we can distinguish between the selective and non-selective form of autophagy. The mechanism of selective autophagy is based on the degradation of specific organelles, such as endoplasmic reticulum (ER), mitochondria, proteasomes, ribosomes, peroxisomes, lipid droplets (LDs), lysosomes and nuclei. Selective autophagy’s mechanism of action is related to the binding of cargoes by autophagy receptors and thereafter its degradation in lysosomes/vacuoles. A distinguishing feature of autophagy receptors is the presence of AIM (Atg8-Interacting Motif) or LIR (LC3-Interacting Region) on their surface. Both of these fragments allow binding of receptors and proteins from Atg8/LC3/GABARAP family selectively [23,24,25,26]. Mentioned proteins are a kind of link between the core autophagic machinery and transported cargo. This enables the selective and efficient recognition of the cargo and its subsequent sequestration in autophagosomes [27]. Based on the types of removed organelles, we distinguish the following subtypes of selective autophagy: ER-phagy (endoplasmic reticulum), mitophagy (mitochondria), proteaphagy (proteasomes), ribophagy (ribosomes), pexophagy (peroxisomes), lipophagy (LDs), lysophagy (lysosomes) and nucleophagy (nuclei) (Figure 2).

The selective autophagy’s ability to remove organelles makes this process a key element in cellular homeostasis maintenance [28]. Disruption of the selective autophagy functions may lead to the occurrence of various disorders, such as cancer [29,30], heart failure [31], metabolic abnormalities [32] and inflammatory [33] or neurodegenerative diseases [34,35].

#### 2.2.1. ER-Phagy

Post-translationally or co-translationally introduced into the endoplasmic reticulum, plasma membrane proteins and secretory proteins inside the ER adopt the native structure. During this process, the newly synthesized polypeptides are often misfolded [36]. Moreover, when mutations occur in the protein-coding sequence, the frequency of this phenomenon increases. To prevent the accumulation of misfolded polypeptides in the ER, they can be transported back to the cytosol and then degraded using the ubiquitin-proteasome system [37,38]. However, proteins may be the only substrates of the mentioned degradation process. In contrast, the autophagy-lysosome system can degrade both protein aggregates and ER membrane lipids. This process is referred to as ER-phagy. There are two basic pathways of ER-phagy: micro-ER-phagy and macro-ER-phagy. In the process of micro-ER-phagy, lysosomal membranes involute and part of the reticulum is “cut off” from the lysosome lumen [39,40].

In contrast, in the macro-ER-phagy process, ER fragments are surrounded by autophagosomes, followed by the fusion of autophagosomes and lysosomes, which results in the formation of autolysosomes where material previously transported by autophagosomes is degraded [41]. Mutations that occurred in *SEC62* and *FAM134B* genes are involved in cancer progression and development, i.e., *FAM134B* mutations in esophageal squamous cell carcinoma promotes tumor development while in colon cancer, it leads to tumor suppression [42,43].

#### 2.2.2. Mitophagy

Mitophagy is the second subtype of selective autophagy. Autophagy machinery recognizes the dysfunctional, obsolete or damaged mitochondria, ultimately leading to the degradation of redundant organelles in lysosomes [44]. Those redundant mitochondria are incapable of efficient oxidative phosphorylation due to their transmembrane potential dissipation. The consequence of this is the reactive oxygen species accumulation and the subsequent increase in oxidative stress level throughout the cell. Specific mitophagy receptors recognize isolated damaged organelles, combine with the core autophagy machinery and leads to the mitochondria-induced ADCD [44,45]. It has been observed that in cancer patients, many proteins involved in mitophagy such as BNIP3, NIX, MFN1 and MFN2 or PINK1 and PINK2 are dysregulated. However, how these proteins interact with cells to act as a tumor promoter (i.e., BNIP3 receptor in pancreatic cancer, melanoma or renal cell carcinoma) or suppressor (i.e., BNIP3 receptor in breast cancer) appears to depend largely on the context and subtype of cancer present in the patient [46].

#### 2.2.3. Proteaphagy

The eukaryotic proteasome, composed of two subunits, regulatory (RP) and core (CP), has an important function in proteostasis maintenance, and by removing e.g., signaling molecules, significantly influences various cellular processes [47]. The role of the RP subunit is to recognize and degrade substrate molecules. The goal of this action is to deliver a target protein to the CP subunit to be degraded [48]. Proteasomes are among the highly mobile complexes, allowing them to move between the cell nucleus and the cytoplasm depending on the phase of the cell cycle, stress conditions or cellular growth [48,49]. In 1995, proteasomes were first observed within lysosomes and autophagic vesicles located in liver cells of starved rats [50]. Twenty years later, in 2015, the term ‘proteaphagy’ was introduced confirming the existence of a proteasome-selective autophagy process [51]. In mammalian cells, the proteasome undergoes amino acid starvation-induced ubiquitination. p62, the autophagy receptor, recognizes these proteasomes, and through the receptor’s concomitant interaction with LC3, they are delivered to the phagophore. In the expanding phagophore, ultimate degradation of the organelles occurs [52]. Despite extensive research, the biological consequences of proteaphagy remain largely unknown. Continued research is needed to determine what role proteaphagy plays in maintaining a population of healthy proteasomes in cells [53].

#### 2.2.4. Ribophagy

Ribosomes represent 10% of the mass of all proteins located in a cell. Their degradation by autophagy is called ‘ribophagy’. In cells in the basal state, the activity of this process is very low, whereas mTOR 1 inhibition or starvation causes an enhancement of ribophagy. Inhibition of mTOR 1 causes transport of NUFIP 1 (Nuclear FMR1 Interacting Protein 1), from the cell nucleus to lysosomes and autophagosomes. Subsequently, NUFIP1 is bound by ribosomes. The degradation of these organelles is initiated by autophagy. Following the interaction between NUFIP1 and ribosomes, LC3 recruits autophagosomes [54,55]. By direct interaction of autophagosomes with LC3, ribosomes are transported to autophagosomes for degradation. To date, little is known about the effects of ribophagy on tumorigenesis. However, the high levels of nucleotides and amino acids in ribosomes suggest that they may provide some sort of nutrient store in the tumor environment [56].

#### 2.2.5. Pexophagy

Peroxisomes are small organelles that degrade lipids in the cytoplasm. Because the estimated half-life of these structures is approximately 2 days, both biogenesis and degradation of peroxisomes are probably dynamic processes [57]. Degradation of peroxisomes by autophagy called ‘pexophagy’, requires the participation of specific autophagy receptors. In the case of pexophagy, these are *NBR1* (a gene adjacent to the *BRCA 1* gene) and sequestosome 1 (*SQSTM1* or *p62*). Overexpression of the above factors induces clustering and subsequent degradation of peroxisomes in mammalian cells [58,59]. As a result of overexpression of ubiquitin molecule-linked PMPs (Peroxisomal Membrane Proteins), such as PEX3 or PMP34, SQSTM1-dependent induction of pexophagy in mammalian cells occurs [60]. Unfortunately, an appropriate answer to the following question remains unknown: “If a PMP is ubiquitinated under pexophagy-inducing conditions and whether subsequent interaction with NBR1 and/or SQSTM1 links ubiquitinated peroxisomes to the autophagic machinery?” [61].

#### 2.2.6. Lipophagy

Lipophagy is the degradation of lipid molecules by autophagy. At the surface of the autophagosome, the interaction of MAP1LC3 (Microtubule-Associated Protein 1 Light Chain 3) with the autophagosomal membrane results in cargo recognition [62]. Lipophagy initiation is usually enabled by the presence of one or more autophagy receptors, e.g., NBR1 or p62, linking the membrane of organelles and LC3 [63]. Depending on the size of the degraded LDs, we distinguish between fragmented microautophagy and macroautophagy. In fragmentary microautophagy, only part of a large lipid droplet is sequestered by autophagosomes. The droplet is then detached as a double-membrane vesicle enriched with LC3 and the contained material is gradually degraded by lysosomes. In contrast, macroautophagy results in the entrapment of the entire lipid droplet inside the autophagosome. After fusion with the lysosome, complete degradation of the droplet occurs in the autolysosome [62,64]. Conducted studies have shown that lipophagy can contribute to both inhibition and stimulation of cancer cell growth. The anti-tumor effect of lipophagy depends on the level of LAL (Lysosomal Acid Lipase), which is a tumor suppressor. Zhao et al. demonstrated that abnormal levels of LAL, specifically a deficiency of this enzyme, enables the growth and metastasis of cancer cells [65]. On the other hand, the carcinogenic effect of lipophagy is related to the possibility of using stored LDs as specific energy resources in the tumorigenesis process, which may contribute to cancer development [66].

#### 2.2.7. Lysophagy

Lysosomes are small, acidic organelles that break down redundant intracellular materials. They contain a large number of hydrolytic enzymes and various membrane proteins. Destabilization of the lysosome leads to the release of significant amounts of hydrolases from its interior into the cytosol, a detrimental phenomenon for the cell [67,68]. Furthermore, lysosome rupture results in the release of calcium ions and protons from the lysosomal compartment into the cytosol, leading to impairment of cellular function [69]. Damaged lysosomes can be degraded by selective autophagy, termed ‘lysophagy’. Following the damage of lysosomal membrane induced by various factors e.g., viral or bacterial toxins, β-amyloid, mineral crystals or lysosomotropic factors the induction of lysophagy occurs [70]. Galectins localized in the cytosol receive signals about the damage that has occurred and induce the ubiquitination of proteins located in the lysosomal membrane. Protein ubiquitination leads to the recruitment of additional adaptors such as SQSTM1. This triggers the core autophagy machinery, engulfment of the damaged organelle by the phagophore, and downstream fusion of normal lysosomes with autophagosomes to degrade damaged lysosomes [71].

#### 2.2.8. Nucleophagy

The last subtype of selective autophagy is degradation of nuclear components, such as RNA, DNA, nuclear proteins or nucleolus, called ‘nucleophagy’. We can distinguish between two types of nucleophagy: macronucleophagy (in mammals) and micronucleophagy (in yeast). Macronucleophagy is based on the degradation of redundant nuclear components via engulfing the material by autophagosomes. Next, autophagosomes merge with lysosomes, where degradation of the redundant material occurs [72,73]. Nucleophagy has a dual function in tumorigenesis—it can both induce and inhibit cancer cells. The carcinogenic effect of nucleophagy, observed in the later stages of tumor growth, is based on the providing of nutrients that allow tumor cells to survive and metastasize in a nutrient-poor environment. In contrast, the anti-cancer effect of this process is based on the removal of damaged DNA or nuclear structures. As a result, it is possible to preserve the normal integrity of nuclear structures and consequently prevent the development of cancer [74].

### 2.3. Microautophagy

The term ‘microautophagy’ was introduced by lysosome discoverer—Christian de Duve. One of the hypotheses put forward by a Belgian scientist, regarding the process of multivesicular lysosomes formation, was “internalization by ‘microautophagy’ of small cytoplasmic buds in shrinking lysosomes” [75]. Microautophagy, a process of non-invasive engulfment of cytoplasmic material through membrane invaginations, occurring directly in lysosomes. Although more than 50 years have passed since the Christian de Duve’s discovery, we still know relatively little about the molecular mechanism of microautophagy as well as how this process is regulated [76].

Mammals’ inability to distinguish between lysosomes and late endosomes results from the complexity of the endocytic system. Furthermore, these structures have the same diameter (about 500 nm) and are significantly smaller compared to autophagosomes found in macroautophagy [77]. All of these aspects make the size of the microautophagic load limited and also the process itself more difficult to detect than macroautophagy [78].

**Figure 3:**
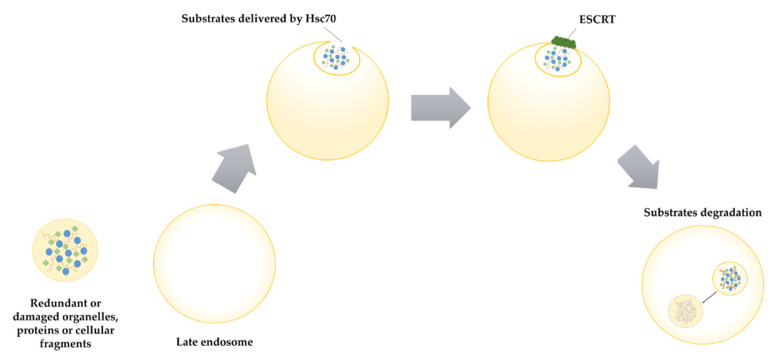
Microautophagy. Redundant or damaged organelles, proteins or cellular fragments are transported inside the formed vesicle to the late endosome by Hsc70. Subsequently, endosomal membrane invagination occurs with the involvement of ESCRT (Endosomal Sorting Complex Required for Transport). The final stage of microautophagy process is the degradation of substrates inside the endosome.

Sahu and co-authors found that in mammalian organisms microautophagy occurs on late endosomes (Figure 3). The substrates of this process are randomly or selectively collected and transported to endosomes in vesicles. Similar to the CMA described above, endosomal microphagy (eMI) substrates have a KFERQ-like motif and are delivered to endosomes by Hsc70 (Heat shock cognate protein 70). However, in contrast to the CMA, eMI process do not require either substrates unfolding or LAMP2A (Lysosome-Associated Membrane Protein type 2A) involvement [79]. Due to the fact that eMI does not require the involvement of LAMP2A, which is found only in avian and mammalian genomes, this mechanism may also occur in other organisms carrying proteins with the KFERQ-like motif [80]. In the eMI process, endosomal membrane invagination occurs with the help of the ESCRT (Endosomal Sorting Complex Required for Transport) machinery [79,81]. Furthermore, another structure which is partially involved in that process is Hsc70, which may cause membrane deformation when bound to phosphatidylserine [79,82]. eMI substrates, integrated into intraluminal vesicles may be degraded in lysosomes/endosomes or can be secreted outside the cell [83].

Mejlvang et al. conducted a study investigating the effect of amino acid starvation on the induction of autophagy. The results showed that starvation leads to immediate activation of autophagic response based on macroautophagy and subsequent eMI. Degradation of autophagy receptors via eMI ensures rapid decomposition of supplied substrates. This enables the maintenance of the initiated anabolic processes and, subsequently, the introduction of appropriate adaptive mechanisms allowing cells to survive a period of prolonged starvation. This phenomenon may be important in the survival and development of cancer cells [84]. To date, endosomal microautophagy is the least studied and described type of autophagy. Its exact role in tumorigenesis remains unclear and requires further studies.

### 2.4. Chaperone-Mediated Autophagy

**Figure 4:**
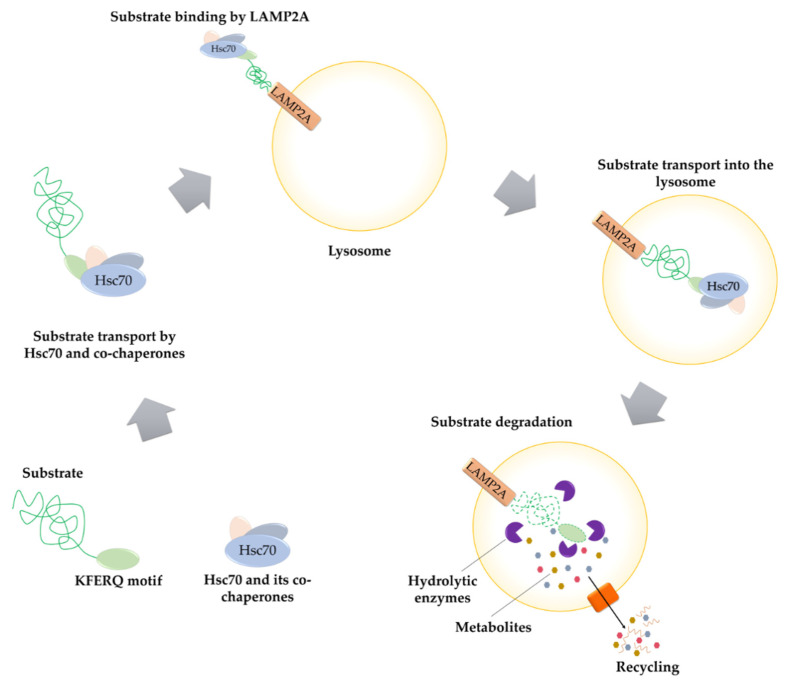
Chaperone-mediated autophagy. Substrate proteins with KFERQ motif are identified by Hsc70 (Heat Shock Cognate protein 70). Next, the recognized substrates are transported by Hsc70 and its co-chaperones on the lysosome surface. Delivered proteins bind to the LAMP2A (Lysosome-Associated Membrane Protein type 2A) and LAMP2A-protein complex is formed. At the final stage of the CMA process, substrate proteins are transported into the lysosome lumen and their degradation by hydrolytic enzyme occurs.

The last of described type of autophagy is chaperone-mediated autophagy (CMA). It is one of the intracellular proteins degradation pathways occurring in lysosomes (Figure 4). Unlike microautophagy, which requires the presence of multilamellar vesicle bodies that capture redundant fragments of cellular organelles [85], in the case of CMA, substrate proteins are identified individually by a cytosolic chaperone, Hsc70. Moreover, the microautophagy process does not require the presence of LAMP2A during cargo transport to the late endosome [85]. CMA selectivity is based on a specific sequence (KFERQ-like motifs) found in all proteins constituting the substrate of that process [86]. Furthermore, in certain cases where the specific motif is incomplete, it is possible to obtain recognizable sequence thanks to post-translational acetylation or phosphorylation [87]. Only the recognized proteins are further transported to the lysosome surface by Hsc70 and its co-chaperones. This mechanism is completely different from that which occurs in microautophagy and macroautophagy processes, where substrates are transported to lysosomes inside the vesicles [11,88]. In the next step, proteins delivered on lysosome surface bind to lysosome-associated membrane protein type 2A. The formation of the protein-lysosomal receptor complex (mass 700 kDa) allows further transport of substrate proteins into the lumen of the lysosome, where hydrolytic enzymes subsequently degrade them [86].

CMA selectivity allows to control the level of many specific proteins in the cell, including proto-oncogenic proteins [11]. The occurrence of CMA dysfunction could lead to the adverse phenomenon of oncogenic protein accumulation inside the cell. One of the important transcription factors is MYC, which level is indirectly regulated by CMA [89]. In CMA-deficient cells, higher levels of MYC are observed. This leads to tumor-beneficial metabolic changes and an increase in the intensity of cell proliferation. Therefore, a normal CMA pathway prevents the malignant transformation of normal cells [89]. Unfortunately, in cancer cells, the anticancer properties of CMA promote tumorigenesis. After transformation, an increase in CMA activity is observed to enable the maintenance of important pro-oncogenic functions [90]. A perfect example of this action is the effect of CMA on hexokinase II, which is a glycolytic enzyme essential for tumorigenesis [91]. As a result of phosphorylation of the enzyme at the Thr473 position, the degradation process of hexokinase II by CMA does not occur, thereby increasing the protein stability. This leads to enhanced glycolysis and stimulates cell growth of HEK293T, MCF-7, MDA-MB-231, and SW480 (breast cancer) lines in vitro and in vivo [92].

## 3. Autophagy and Programmed Cell Death—Double-Edged Sword Relationship

**Figure 5:**
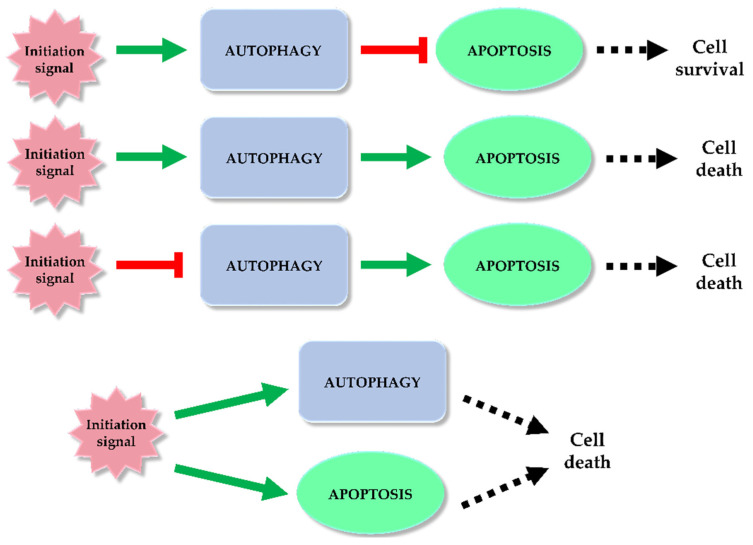
Autophagy and apoptosis relationship. Among the potential correlation pathways between autophagy and programmed cell death we can distinguish: activation of autophagy and apoptosis inhibition, activation of autophagy and activation of the apoptotic pathway, autophagy suppression and induction of apoptosis or simultaneous activation of autophagy and apoptosis leading to ADCD and apoptosis. The inhibitory effect of each process (red mark) and inducting effect (green mark) is indicated on the scheme.

Autophagy and apoptosis are regulated in the cell by different mechanisms. However, it happens that both processes overlap. Under the influence of stress, sequential or simultaneous activation of the apoptotic and autophagy pathways can occur in a cell. There are potential pathways of the relationship between apoptosis and autophagy: activation of autophagy and subsequent inhibition of apoptosis, activation of autophagy leading to activation of the apoptotic pathway, autophagy suppression and induction of apoptosis or activation of autophagy and apoptosis simultaneously, leading to cell death on apoptotic and autophagy-dependent pathway (Figure 5) [93,94,95]. In the former case, the cell activates autophagy in response to a stress signal. As a defense mechanism, autophagy leads to the removal of damaged fragments, preventing the activation of the apoptotic pathway. The second possibility is a situation in which the cell is no longer able to defend itself against the resulting damage, and the activated autophagy subsequently leads to activation of apoptosis and cell death. In the last case, a stress signal triggers both processes, resulting in cell death via two pathways [93]. Key factors connecting apoptosis and autophagy include, for example: p53, Bcl-2/Beclin 1, Atg proteins, p62 or caspases.

### 3.1. p53 in Apoptosis and Autophagy

p53, a protein which binds specific DNA sequences, is involved in many cellular processes including repair of damaged DNA and induction of apoptosis. Due to its ability to regulate the cell cycle, p53 is called the guardian of the genome [96]. Activation of this factor can occur, for example, as a result of DNA damage, hypoxia, or nutritional stress [97,98,99]. p53 can affect both the extrinsic and intrinsic pathway of apoptosis. DNA damage causes mitochondrial translocation of p53. The protein promotes cytoplasmically localized Fas and TRAIL receptors, leading to induction of the extrinsic apoptotic pathway [100,101]. However, in the cell nucleus, p53 promotes many proapoptotic proteins, such as Bid, PUMA or Bax. In addition, it leads to inhibition of Bcl-2 expression, and both of these actions trigger the intrinsic apoptosis pathway [101].

The p53 protein is also involved in the regulation of autophagy. Based on their study, Crighton and co-authors found that genotoxic stress results in transcriptional activation of DRAM (Damage-Regulated Autophagy Modulator), a direct target gene of p53, which causes induction of autophagy. The DRAM signaling cascade promotes the fusion of autophagosomes and lysosomes, resulting in the formation of autolysosomes. This p53 target gene is an essential factor in the proper functioning of the apoptosis regulatory network and p53-dependent autophagy [102]. Furthermore, Tasdemir et al. demonstrated that cytoplasmically localized p53 through inactivation of AMPK and subsequent activation of the mTOR signaling pathway leads to inhibition of autophagy in the cell [103].

Scherz-Shouval and co-authors detected a relationship between autophagy and apoptosis processes. They revealed that under starvation conditions, p53 post-translationally inhibits the regulation of LC3 level, which leads to its accumulation in cells and decreases the rate of the autophagy process. The consequence is cell death by apoptosis [104].

### 3.2. Bcl-2/Beclin 1 in Apoptosis and Autophagy

Bcl-2, members of the B-cell lymphoma family of proteins, inhibits the release of cytochrome c from the mitochondrial interior, thereby playing a key role in the intrinsic apoptotic pathway [105]. Beclin 1 is a key element involved in autophagosome formation and is also an important component of the PI3K/Vps34 class III complex [106]. Bcl-2 binding to Beclin 1 leads to dissociation of Beclin 1 from PI3K class III, which results in inhibition of autophagy [107]. However, the occurrence of mutation in the BH3 receptor domain of Bcl-2 or Beclin 1 domain leads to dysfunction of Bcl-2/Beclin 1 complex, intensification of autophagy and promotion of cell survival [108,109].

Under nutrient-deficient conditions, autophagy is an essential element for cell survival. Activation of JNK1 (C-Jun N-terminal protein Kinase 1) and phosphorylation of residues involved in the Bcl-2′s regulatory loop lead to the destruction of the Bcl-2/Beclin 1 complex and consequently to initiation of autophagy [110]. Under standard conditions the phosphorylated Bcl-2 molecule binds to Bax, leading to inhibition of apoptosis. Due to the normal phosphorylation of Bcl-2, it is possible to maintain the integrity of the mitochondrial membrane, which in turn protects cells from death by the intrinsic apoptotic pathway. Sustaining the integrity of the mitochondrial membrane prevents the release of proapoptotic proteins from within the organelle into the cytoplasm [111]. However, in the situation of long-term nutrient deficiency, autophagy is not able to alleviate cellular damages. Intensification of Bcl-2 phosphorylation (hyperphosphorylation) promoted by JNK1 occurs [112]. This results in dissociation of the Bcl-2 molecule from Bax and apoptotic cell death. When the cell receives adequate amounts of nutrients, Bax/Bak and Beclin 1 bind to Bcl-X_L_ or Bcl-2, leading to the inhibition of activation of both processes, apoptosis and autophagy [109,113].

### 3.3. Atg Proteins in Apoptosis and Autophagy

The level of autophagy-related proteins in a cell is regulated by the availability of growth factors and nutrients essential for proper cell functioning. Among Atg proteins we can distinguish the Atg12–Atg5 complex, which is important in both autophagy and apoptosis [114].

The Atg12–Atg5 complex, essential for autophagosome formation, also participates in the apoptotic pathway in an unconjugated form. Atg12 binding through a BH3-like motif to Bcl-2 and Mcl-1 (Myeloid Cell Leukemia 1) increases the intensity of the intrinsic apoptotic pathway. Interestingly, the anti-apoptotic properties of Mcl-1 can be inhibited in the cell as a result of abnormal Atg12 expression. Moreover, silencing Atg12 in an apoptotic cell will result in the inhibition of Bax induction and the arrest of cytochrome c release from the mitochondrion [115]. Cleaved by cell stress-activated cysteine proteases (caplains), Atg5 plays a significant role in the initiation of the intrinsic apoptosis pathway. As a consequence of cleavage, translocation of the N-terminal part of the Atg5 protein into the mitochondrion occurs. Inside the organelle, this fragment interacts with Bcl-X_L_ allowing Atg-5 to be involved in the release of cytochrome c from the mitochondrion and indirectly participating in apoptosis promotion [116]. Taken together, Atg5 and Atg12 proteins may be involved in both autophagy and apoptosis, depending on the cellular conditions.

### 3.4. p62 in Apoptosis and Autophagy

p62, also known as SQSTM1, is a multi-domain adaptor protein that controls cell viability by regulating both autophagy and apoptosis [117]. By polymerizing with other p62 molecules, this protein has the ability to accumulate ubiquitin-tagged proteins. Aggregates of p62 (called p62 speckles), through their storage properties and ability to bind to the LC3 molecule, recognize, gather, and most importantly transport cargo to the autophagosomes [96]. p62, through its ability to activate caspase-8 on the autophagosome membrane also plays an important role in the induction of apoptosis. The autophagy-dependent mechanism of caspase-8 activation involves simultaneous induction of autophagy and activation of caspase-8. The autophagosomal membrane provides some kind of platform on which the caspase cascade leading to cell death by apoptosis is initiated. Depletion of Atg3 or Atg5 leads to the suppression of autophagosome formation, which in turn results in the inhibition of caspase-8 activation and subsequent suppression of apoptosis [118].

### 3.5. Caspases in Apoptosis and Autophagy

Caspases, enzymes belonging to the group of cysteine proteases, have been known to science for a long time. Their participation and the exact mechanism of action in the process of apoptosis have been widely studied and described in many scientific articles [119,120,121]. These enzymes are involved in both intrinsic and extrinsic pathways of apoptosis, acting as initiators (caspases-2, -8, -9 and -10) or effectors (3, -6 and -7) [122]. Caspases under standard conditions occurring in the form of inactive zymogenic precursors can be activated under the influence of various external or internal stimuli that initiate apoptosis. Activated enzymes may participate in the apoptotic pathway [123]. Despite the significant differences between the autophagy and apoptosis processes, the conducted studies indicate that caspases also affect the autophagy process. Oral and co-authors have shown that overexpression of caspase-8 leads to degradation of Atg3 protein and thus prevents its pro-autophagic activity [124]. Furthermore, Wirawan et al. showed that two key components of the autophagy-inducing complex (class III PI3K and Beclin 1) are direct substrates of caspases. It was observed that in response to different signals inducing the two apoptotic pathways, these enzymes cause cleavage of the complex components. Thus, the researchers confirmed that class III PI3K and Beclin 1 are substrates of caspases [125]. In contrast, Han and co-authors showed that caspase-9, by promoting Atg7-dependent LC3-II transformation, facilitates autophagosome formation. Moreover, the authors showed that depending on the cellular conditions, Atg7 can also form a complex with caspase-9 and directly inhibit the proapoptotic activity of the enzyme [126]. All of these studies indicate there is a mutual correlation between autophagy and apoptosis processes.

## 4. Autophagy Inhibitors and Activators

**Figure 6:**
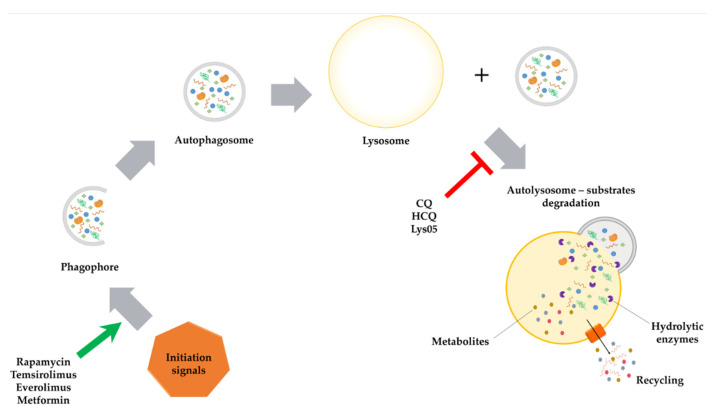
The influence of selected inhibitors or activators on autophagy process. Rapamycin, temsirolimus, everolimus and metformin via inhibition of initiation signals, stimulates autophagy process (marked with green arrow on the scheme). Chloroquine (CQ), hydroxychloroquine (HCQ) and Lys05 through blocking of autophagosome and lysosome fusion inhibits the autophagy (marked with red T-shaped sign on the scheme).

In a cancer therapy context, autophagy is a dichotomous process—it may inhibit or induce tumor growth (Figure 6) [127]. As a mechanism that promotes cancer cells development, autophagy protects cells from the negative impact of various forms of cellular stress. In anti-cancer therapy, that process is referred to as ‘adaptive autophagy’. It sustains cancer cells growth, increasing chances of tumor survival despite the use of toxic chemotherapeutics or ionizing radiation. However, intentional inhibition of adaptive autophagy leads to reversal of this phenomenon, causing cells re-sensitization to ionizing radiation or used chemotherapeutic agents [128,129]. On the other hand, autophagy can promote genomic stability and inhibits inflammation at the early stage of carcinogenesis process. Interestingly, in animals disruption of *ATG* genes results in accelerated cancer development [128].

### 4.1. Autophagy Inhibitors Undergoing Clinical Trials

#### 4.1.1. Chloroquine

Chloroquine (CQ) is a compound known for many years. This aminoquinolone derivative was first approved by the U.S. Food and Drug Administration (FDA) in October 1949 as an antimalarial agent [130]. Although more than 70 years have passed since the CQ was discovered, the detailed mechanism of the antimalarial effect of this agent remains unknown. Presumably, CQ as a weak base, acting as a lysosomotropic compound, inhibits lysosome activity [131]. Chloroquine is protonated after entering the lysosome, due to low pH inside the organelle. Protonated CQ accumulation inside the lysosome leads to inhibition of autophagic cargo degradation and consequently blocked autophagic flux [132]. Inhibition of charge degradation located inside the lysosome stops the last autophagy stage. As a consequence, the ability to provide energy to the cell through the autophagy process is blocked. CQ’s ability to inhibit autophagy is being used by scientists e.g., in the investigation of new cancer therapy methods.

Erkisa et al. [35] published an article describing the combination therapy of metastatic prostate cancer using the palladium(II) barbiturate complex and CQ. The author’s study showed increased efficacy of combined therapy: CQ and palladium(II) barbiturate complex compared to single-agent (CQ or palladium(II) barbiturate complex alone) treatment. The use of CQ resulted in inhibition of prosurvival autophagy function and consequently increased the sensitivity of tumor cells to the tested complex [133]. A paper recently published by Lopiccolo and co-authors describes in vitro and in vivo studies using chloroquine and nelfinavir as a combination therapy in non-small cell lung cancer (NSCLC) treatment. The obtained results indicate that both in vitro and in vivo, combination therapy was effective in NSCLC treatment. The combined administration of CQ and nelfinavir resulted in increased inhibition of NSCLC cell growth while enhancing apoptosis and ER stress induction [134]. Next interesting, this year’s paper is an article published by Wei et al. describing the use of cyanidin-3-O-glucoside (C3G) combined with CQ in *Drosophila* malignant Raf^GOF^scrib−/− model to determine the antitumor activity of C3G. Results presented in the paper revealed that CQ and C3G combined therapy is more effective against *Drosophila* malignant Raf^GOF^scrib−/− model that CQ or C3G used alone [135]. All papers and results mentioned above suggest that the combination of CQ with the different compound may be more effective than single-agent therapy.

#### 4.1.2. Hydroxychloroquine

Hydroxychloroquine (HCQ), belonging to the 4-aminochinoline class, is a CQ analogue. The original CQ molecule has been enriched with a hydroxyl group, thus forming HCQ, which compared to the parent compound is three times less toxic [136]. In 1955, HCQ was approved by the FDA and, like CQ, registered as an antimalarial agent [137]. Hydroxychloroquine as an inhibitor of autophagy process blocks autolysosomes formation by preventing lysosomes and autophagosomes fusion [138,139]. Both HCQ and described above CQ have been used as standard autophagy inhibitors in many clinical and preclinical studies. Only right now (May 2021) there are at least a dozen active clinical trials on the use of HCQ in the treatment of various cancers (ClinicalTrials.gov, accesses on 27 May 2021). The Emory University is actively recruiting patients for a trial investigating the use of HCQ in combined therapy (HCQ + paricalcitol with standard chemotherapeutics: gemcitabine + nab-paclitaxel) of metastatic or advanced pancreatic cancer (NCT04524702). As another example, M.D. Anderson Cancer Center is investigating the use of HCQ with letrozole and palbociclib in patients with estrogen receptor-positive, HER2 negative breast cancer before they undergo surgery. This study aims to enhance the efficacy of the provided treatment (NCT03774472). Finally, it is also worth mentioning that there are many ongoing clinical trials on the use of CQ and HCQ in the treatment of patients with COVID-19 (ClinicalTrials.gov).

#### 4.1.3. Verteporfin

Verteporfin is benzoporphyrin derivative consisting of two regioisomers (I and II). This compound was approved by the FDA in 2002 for photodynamic therapy of patients with age-related macular degeneration [140,141]. To find new autophagosomes accumulation inhibitors, scientists decided to screen the databases of off-patents agents and libraries of compounds with known pharmacological activity. Among ≈3500 of screened compounds, only verteporfin (VP) was selected for further investigation. Donohue et al. examined the ability of verteporfin to inhibit autophagy process by pre-treating MCF-7 cells with CQ. Autophagosomes accumulation induced by CQ was subsequently inhibited by verteporfin. Furthermore, inhibition of accumulation of autophagosomes occurred in the dark. Based on this, the authors concluded that the ability of verteporfin autophagy inhibition is not associated with its photodynamic properties [141]. Researchers are investigating the use of verteporfin in the treatment of various cancers. The increased efficacy of gemcitabine in combination with verteporfin in the treatment of pancreatic ductal adenocarcinoma model, the improved effectiveness of sorafenib therapy with VP against hepatocellular carcinoma or the increased sensitivity of osteosarcoma cells to treatment caused by the use of VP have been demonstrated [142,143,144]. In addition, currently ongoing clinical trials are investigating the use of VP, e.g., for the treatment of recurrent prostate cancer (NCT03067051) or pancreatic cancer therapy (NCT03033225).

#### 4.1.4. Clarithromycin

Clarithromycin (CAM) is well-known medicine, belonging to the class of macrolide antibiotics. Approved in 2000 by the FDA [145], CAM is commonly used in therapy of various bacterial infections, treatment of *Helicobacter pylori*-induced gastric infections or Lyme disease therapy. Data collected from the extensive clinical and preclinical studies on CAM indicate that the drug, combined with conventional therapeutics, could be used to treat various cancers. CAM’s anticancer properties are based on its ability to anti-angiogenesis, pro-inflammatory cytokines reduction and autophagy inhibition [146]. After the fusion of autophagosomes and lysosomes, autophagy is blocked by inhibition of lysosomes function [147]. Ongoing clinical trials are investigating the CAM’s application in the treatment of: multiple myeloma (NCT04302324, NCT04063189, NCT02542657), mucosa-associated lymphoid tissue lymphoma (NCT03031483) and previously untreated, advanced-stage indolent lymphoma (NCT00461084).

Information regarding the compounds undergoing clinical trials is collected in Table 1. Target points, adverse effects and selected therapeutic schemes of described autophagy inhibitors are presented in Table 2.

### 4.2. Autophagy Inhibiotors Undergoing Preclinical Trials

#### 4.2.1. 3-Methyladenine

3-Methyladenine (3-MA) was discovered in 1982 by Seglen & Gordon. The scientists through screening of a large number of N6-methylated adenosine derivatives selected the most promising compound, which appeared to be 3-MA [150]. Nowadays, 3-MA is one of the most commonly used autophagy inhibitor [151]. This compound affects two molecular targets involved in the autophagy process: phosphoinositide 3-kinase (PI3K) and Vps34. The duality of the compound’s action means that it affects autophagy with increased potency [152,153]. Wu et al. in their work described the duality of 3-MA action. Based on the obtained results scientists concluded that the compound, when administered over a prolonged period, in nutrient-rich conditions, promotes autophagic flux. However, under nutrient-deficiency conditions, it inhibits autophagy [154]. 

Scientists around the world are conducting research on the use of 3-MA combined with different drugs in the therapy of various cancers. Wang et al. showed in their in vitro studies that resveratrol used alone against human ovarian serous papillary cystadenocarcinoma cell line SK-OV-3 can inhibit apoptosis by inducing autophagy. Furthermore, results obtained from combined therapy (resveratrol with 3-MA) revealed that simultaneous application of autophagy inhibitor and chemotherapeutic drug in SK-OV-3 tumor could improve the drug efficiency and also protect normal cells from tumorigenesis [155]. In a recently published paper, Zhao et al. investigated the effect of 3-MA on the treatment of hepatocellular carcinoma cells (HepG2 cell line). They showed that 3-MA (used in combined therapy with sorafenib), by inhibiting the autophagosome formation, leads to a reduction of acquired sorafenib resistance of HepG2 cells [156].

#### 4.2.2. SAR405

SAR405, Vps34 and Vps18 inhibitor with low molecular mass, was first described in 2014 by Ronan et al. [157]. Research published a year after by Pasquier revealed that inhibition of Vps34 by SAR405 leads to the impairment of lysosome function and inhibition of autophagy process [158]. In 2020, Janji et al. published an article describing the usage of Vps34 inhibitors (SAR405 and SB02024) in the therapy of colorectal and melanoma tumor cells. Based on the obtained results, scientists concluded that the use of these compounds enhances the therapeutic effect of the applied anti-PD-1/PD-L1 immunotherapy [159].

#### 4.2.3. Lys05

Lys05 is a water-soluble bisaminoquinoline inhibitor of autophagy. The enhanced autophagy inhibition by Lys05 compared to CQ and HCQ is attributed to the presence of C-7 chlorine, triamine linker and two aminoquinolone rings in the Lys05 structure. A study conducted by McAfee and co-workers compared the efficacy of HCQ and Lys05 in treatment of C8161, PC-9, LN-229 cell lines and 1205Lu xenograft model. Obtained in vivo results revealed 34-fold higher Lys05 concentration in tumor cells compared to HCQ. Moreover, a Lys05 application-related double accumulation of autophagy vesicles compared with HCQ therapy in a used xenograft model was observed [160]. 

DeVorkin et al. published an article in which they showed that the administration of Lys05 together with sunitinib (receptor tyrosine kinase inhibitor) improve the therapeutic effect of this drug. In used clear cell ovarian carcinoma xenograft models, inhibition of autophagy process by Lys05 resulted in enhancing the anti-cancer activity of sunitinib compared with single-agent treatment (sunitinib or Lys05 alone) [161]. In an article titled, “Targeting quiescent leukemic stem cells using second generation autophagy inhibitors,” Baquero et al. investigated the potential application of Lys05 with tyrosine kinase inhibitors in the treatment of chronic myeloid leukemia (CML). The obtained results showed that Lys05-mediated inhibition of autophagy process affects tumor cells via reduction of quiescence of leukemic stem cells and increasing the expansion of myeloid cells [162].

#### 4.2.4. ROC-325

ROC-325 is a compound developed by applying a logical medicinal chemistry approach to drug design. To create a more effective, well-tolerated and more potent autophagy inhibitor, scientists generated new dimeric compounds based on the modified CQ, HCQ and lucanthone (antischistosomal drug) elements. Carew et al., based on the obtained results, concluded that ROC-325 (with lucanthone and HCQ motifs) exhibited significantly greater anti-cancer activity against various types of cancer than the parent compounds. Moreover, they found that ROC-325 used at much lower doses inhibited autophagy more effectively than HCQ. 

Based on in vitro studies using renal cell carcinoma models, it was possible to determine the ROC-325 effect on the autophagy process ROC-325 was shown to inhibit autophagic flux as well as lead to the autophagosomes accumulation and lysosomes deacidification. Under in vivo conditions, the compound administered orally to mice at low doses inhibited tumor growth more effectively than HCQ administered at high doses. Furthermore, by analyzing of tumor samples from ROC-325 treated mice, autophagy was inhibited and apoptosis and proliferation of tumor cells were reduced [163]. In 2019, Nawrocki et al. conducted preclinical in vitro and in vivo studies on the use of ROC-325 in acute myeloid leukemia (AML). The in vitro studies examined the efficacy of ROC-325 (single-agent treatment or in combination with azacitidine) against four tumor cell lines: MV4-11, MOLM-13, KG-1 and HL-60. During in vivo studies, mice were treated with azacitidine (AZA), ROC-325 or AZA + ROC-325 combination. The results obtained in vitro as well as in vivo indicated that combination therapy is more effective and significantly extended the overall survival time. In addition to this, the combined agents were well tolerated [164].

#### 4.2.5. Spautin-1

Spautin-1 was originally identified as a selective and strong phosphodiesterase type 5 inhibitor [165,166]. During preclinical studies, the researchers discovered that spautin-1 is also an autophagy inhibitor. The obtained results revealed that by promoting the degradation of Vps34 complexes, spautin-1 inhibits two ubiquitin-specific peptidases (USP10 and USP13), which consequently leads to inhibition of the autophagy process [167]. The collected data suggest that spautin-1 could be used in the treatment of ovarian cancers [168], CML [169] or prostate cancer [170].

#### 4.2.6. MM124 and MM137

MM124 and MM137 are compounds belonging to a group of 7-methyl-5-phenyl-pyrazolo[4,3-*e*]tetrazolo[4,5-*b*][1,2,4]triazine sulfonamide derivatives. In a study conducted by Gornowicz et al., the anticancer effects of new derivatives on colorectal cancer cells were investigated. The obtained results showed that MM124 and MM137 decrease the concentration of LC3A, LC3B and Beclin 1 in the tested cell lines (DLD-1 and HT-29). Based on this, the researchers concluded that MM124 and MM137 could inhibit the autophagy process at the autophagosome formation level. Nevertheless, further in vivo studies are required to confirm the autophagy-inhibiting effect of the novel derivatives [171].

Table 3 summarizes information about autophagy inhibitors undergoing preclinical investigation.

### 4.3. Autophagy Activators Undergoing Clinical Trials

#### 4.3.1. Rapamycin

Rapamycin (RAPA, Sirolimus) is a 31-membered macrocyclic antifungal antibiotic produced by *Streptomyces hygroscopicus* [182]. This naturally occurring mTOR inhibitor was first approved by the FDA in 1999 as Sirolimus [183]. RAPA is the compound with a broad spectrum of pharmacological and biological activity. In addition to its antifungal activity, this compound exhibits e.g., neuroprotective [184], antitumor [185], anti-ageing [186] and immunosuppressive properties [187]. mTOR signaling plays a crucial role in autophagy occurring in cancer cells by increasing their growth and enhancing proliferation. Inhibition of mTOR activity induced by RAPA may cause an increase in autophagy flux in tumor cells and consequently contribute to a reduction in tumor growth. Furthermore, conducted studies revealed that RAPA induces autophagosomes formation and, at the later stage, lysosomes and autophagosomes fusion [188,189,190]. Ongoing clinical trials, concerning the application of RAPA in the treatment of, e.g., kaposiform hemangioendothelioma in children (NCT04077515), bladder cancer (NCT02753309, NCT04375813), HER2+ metastatic breast cancer (NCT04736589) and refractory solid tumors (NCT02688881). There are also ongoing studies investigating the use of sirolimus in combination with durvalumab for the treatment of NSCLC (NCT04348292) and sirolimus with metronomic therapy for the treatment of pediatric relapsed or refractory tumors (NCT02574728).

#### 4.3.2. Temsirolimus

Temsirolimus (CCI-779, TEM, Torisel^®^) is a known analogue of RAPA. This water-soluble RAPA’s prodrug was first developed by Wyeth Pharmaceuticals and it was approved by the FDA in the treatment of advanced renal cell carcinoma (RCC) [191] in 2007. TEM is produced via RAPA and 2,2-dihydroxymethylpropionic acid esterification [192]. However, due to the ease of the degradation of orally administered esters, this drug must be administered intravenously [193]. Studies on the potential use of TEM in the therapy of colorectal cancer [194], prostate cancer [195], human papillomavirus-related oropharyngeal squamous cell carcinoma [196] or advanced solid tumors [197] have been conducted. Furthermore, there are at least several dozen ongoing clinical trials on the use of temsirolimus for the treatment of advanced or metastatic malignancies (NCT01552434), advanced gynecological malignancies (NCT01065662), advanced rare tumors (NCT01396408), diffuse intrinsic pontine glioma (NCT02420613) or solid tumors in adults (NCT01375829) are under investigations.

#### 4.3.3. Everolimus

Everolimus (RAD001) is a next RAPA water-soluble analogue, developed by Novartis. The drug is produced via the esterification (ethylene glycol plus RAPA) process. Compounds esterification results in the formation of a new derivative (RAD001) with improved solubility in water and stability [93]. Everolimus was first approved by the FDA in 2009 as a therapeutic agent in the treatment of advanced renal carcinoma [198]. Since then, the new therapeutic applications of the drug in the treatment of various cancers have been continuously developed. In the past year alone, the possibility of using RAD001 in various cancer therapies has been investigated, e.g., in combination therapy (everolimus plus bevacizumab) for advanced papillary variant renal cell carcinoma [199], in the treatment of triple-negative breast cancer (everolimus in combination with gefitinib) [200] or in the treatment of advanced solid tumors (everolimus plus vatalanib) [201]. Moreover, there are current, ongoing clinical trials concerning the application of RAD001 on the treatment of, e.g., recurrent or progressive ependymoma in children (NCT02155920), Hodgkin lymphoma (NCT03697408), metastatic transitional cell carcinoma of the urothelium (NCT00805129), advanced gynecologic malignancies and breast cancers (NCT03154281) or recurrent low grade gliomas in young adults and pediatric patients (NCT04485559).

#### 4.3.4. Metformin

Metformin was discovered in 1922 as a by-product of the synthesis of N,N-dimethylguanidine [202]. As a result of numerous studies, the hypoglycemic effect of metformin was discovered, and it was first used in the treatment of diabetes in 1957 [203]. Nowadays, this compound, approved by the FDA in 1998, is the most commonly prescribed antidiabetic drug and is used in the treatment of type 2 diabetes, especially in obese diabetics [204,205,206]. Based on the conducted studies, Tomic et al. were found that metformin significantly affects melanoma cells proliferation by inhibiting tumor growth. Moreover, based on the tumor samples analysis the drug was found to increase the level of apoptosis markers in cancer cells and induce the autophagy process [207]. In 2014, Takahashi and co-authors obtained similar results during a study with endometrial cancer cells [208]. Recently, intensive studies exploring new properties of this compound were conducted. The potential use of metformin, for example in the treatment of PCOS [209,210], in cancer therapy [211], as a cardiovascular protector [212] or as an inhibitor of the ageing process [213,214], has been investigated. There are many ongoing studies on the use of metformin in cancer treatment. Currently, clinical studies are being conducted on the use of the compound in the treatment of, for example, breast cancer (NCT04559308, NCT04387630, NCT01980823, NCT04741204), colon cancer (NCT03359681), thoracic neoplasm (NCT03477162) or prostate cancer (NCT02176161, NCT02339168).

Described autophagy activators undergoing clinical trials and their target points, adverse effects or selected therapeutic schemes are listed in Table 4 and Table 5.

### 4.4. Autophagy Activators Undergoing Preclinical Trials

#### 4.4.1. Miconazole

Miconazole (MCZ), an imidazole derivative, is a known antifungal drug originally approved by the FDA for the treatment of vaginal candidiasis in 1974 [215]. Moreover, this drug is used also in the treatment of athlete’s foot [216] or tinea versicolor [217]. Interestingly, in recent decades MCZ has attracted scientist as a potential drug with anti-cancer properties. Conducted studies have shown, that MCZ inhibits the growth of various human tumors, e.g., breast cancer and glioma [218,219] or osteosarcoma [220]. Jung et al. have been investigated the effect of MCZ on the autophagy process. Conducted studies revealed that MCZ induces autophagy in glioblastoma cells. The authors presumed, that MCZ-induced autophagy-mediated cell death might be activated via reactive oxygen species-mediated endoplasmic reticulum stress [221]. In published recently paper, Ho et al. have shown that MCZ induces autophagy process in bladder cancer cells. The authors demonstrated that miconazole increases the autophagic flux and promotes the expression of LC3 in the tested cancer cells. They revealed that combination therapy (MCZ with autophagy inhibitor) enhanced the anticancer properties of miconazole [222].

#### 4.4.2. CRO15

CRO15 is a new compound derived from metformin, recently identified by Jaune and co-authors. The research aimed to develop a new molecule with a better pharmacological profile, enhanced potency and improved effect in patients compared to its parent drug, metformin. The initial screening and structure-activity relationship studies revealed a new potential drug—CRO15. Extensive in vitro, in vivo studies and studies in melanoma xenograft models have shown that CRO15 reduces tumor cell viability. The molecular mechanism of action of the compound is based on effects on two main processes—autophagy and apoptosis. The results obtained, both in vitro and in vivo, showed that CRO15 induces autophagy by accumulating LC3 in melanoma cancer cells. Moreover, the performed in vivo studies did not show strong toxicity of the tested compound in mice. All of this data suggests that CRO15 should be further evaluated as a potential anticancer drug [223].

#### 4.4.3. α-Hederin

α-Hederin (α-HN) is a molecule belonging to the wide group of monodesmosidic triterpenoid saponins. This compound is the main component isolated from *Hedera helix L.* leaves. It is also found in *Nigella sativa*, *Kalopanax pictus* and *Chenopodium quinoa* plants [224]. Studies conducted by Li et al. revealed that α-HN may act through increasing the ROS concentration, consequently leading to the activation of the intrinsic apoptotic pathway [225]. This finding prompted Li and co-workers to investigate the influence of α-HN on the autophagy process in colorectal cancer cells. Obtained results have shown that α-HN induces autophagy-mediated cell death through the activation of the ROS-dependent AMPK/mTOR signaling pathway. Nevertheless, the potential use of α-HN as an anticancer agent requires further investigation due to its toxicity, hemolytic effect and protein absorption [226].

#### 4.4.4. MJ-33

MJ-33 is a novel quinazolinone derivative synthesized by Ha and co-authors. A recently published paper revealed the anti-cancer properties of this compound in 5-fluorouracil-resistant (5FUR) colon cancer cells (HT-29/5FUR). Furthermore, the molecular mechanism of MJ-33 activity was also investigated. Based on the obtained results, MJ-33 was found to induce the autophagy process in HT-29/5FUR cells through inhibition of mTOR phosphorylation and subsequent upregulation of ATG proteins expression. Additionally, combined therapy with MJ-33 and known autophagy inhibitor, 3-MA, has shown significant enhancement in effector caspases (caspase-3 and caspase-7) activity compared with single-agent therapy with MJ-33. Obtained results suggest that the autophagy process plays a cytoprotective role in tested HT-29/5FUR cells [227]. Nowadays, scientists around the world investigate the effect of combined therapies, autophagy inhibitors together with autophagy activators, as a novel strategy in cancers treatment [228]. The authors of the aforementioned paper suggest that further studies on new quinazolinone derivative should examine the effect of combined therapy with MJ-33 and autophagy inhibitors [227].

Table 6 summarizes information about all described autophagy activators undergoing preclinical investigation.

## 5. Conclusions

Neoplastic transformation requires significant changes in biological processes as part of increased demand and consumption of energy under stressful conditions. It leads to intracellular adaptation that ensures survival in conditions with a limited amount of nutrients and oxygen. There is a change in metabolism, protein and organelle turnover, and bioenergy functions. These neoplastic signaling pathways cross with autophagy at many levels. Autophagy is a dichotomous process—it may inhibit or induce tumor growth. These observations suggest that autophagy plays a dynamic and complex role play in cancer, which may, in fact, explain the duplicity of autophagy in carcinogenesis. While targeting autophagy pathways appears to be a promising tool in developing new anti-cancer therapies, recent findings suggest that the underlying molecular mechanisms and specific targets of autophagy in cancer need to be well defined before it can be used effectively in pharmaceutical and medical research.

Despite the fact that the most well-known inhibitors (such as chloroquine, hydroxychloroquine, clarithromycin or verteporfin) and activators (rapamycin, metformin, temsirolimus or everolimus) of autophagy are recognized in the scientific and medical world for years, they have not been used in medicinal practice. As presented in this paper, a number of clinical and preclinical studies are conducted, the aim of which is to discover new possibilities in oncological therapy, including the use of autophagy modulators in combination with anticancer drugs. Recent studies have identified new classes of inhibitors and activators of autophagy that are currently in preclinical research. Among them, the most promising are 3-Methyladenine, SAR405, Lys05, 7-methyl-5-phenylpyrazolo[4,3-*e*]tetrazolo[4,5-*b*][1,2,4]triazine sulfonamide derivatives, miconazole, CRO15 or α –Hederin. These compounds have different target points in the autophagy process and further detailed studies are needed to determine their potential use in the practical treatment of cancer.

## Figures and Tables

**Table 1:** Selected autophagy inhibitors under clinical investigation.

Autophagy Inhibitor	Chemical Structure	Study Type	References
Chloroquine	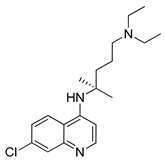	**Preclinical studies:** metastatic prostate cancer, NSCLC treatment	[133,134,135]
Hydroxychloroquine	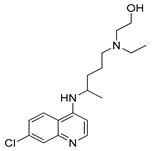	**Clinical trials:** therapy of metastatic or advanced pancreatic cancer or HER2 negative breast cancer	NCT04524702, NCT03774472
Verteporfin	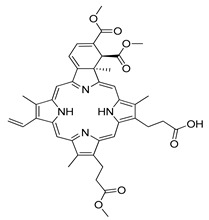	**Preclinical studies:** treatment of pancreatic ductal adenocarcinoma, hepatocellular carcinoma or osteosarcoma **Clinical trials:** recurrent prostate cancer or pancreatic cancer treatment	[141,142,143,144] NCT03067051, NCT03033225
Clarithromycin	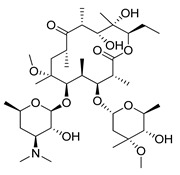	**Clinical trials:** multiple myeloma, mucosa-associated lymphoid tissue lymphoma or previously untreated, advanced-stage indolent lymphoma therapy	NCT04302324, NCT04063189, NCT02542657, NCT03031483, NCT00461084

**Table 2:** Target points, adverse effects and selected therapeutic schemes of autophagy inhibitors.

Autophagy Inhibitor	Target Point	Adverse Effects	Therapeutic Combination or Single-Agent Treatment	Tumor Type	References
Chloroquine	lysosomes	headache, visual disturbances, pruritus or gastrointestinal upset, the risk of acute kidney injury due to kidney cells sensitization to chemotherapy [148]	CQ + temozolomide + radiation therapy	glioblastoma, gliosarcoma and astrocytoma (grade IV)	NCT04397679, NCT02432417
			CQ + taxane after anthracycline failure	advanced or metastatic breast cancer	NCT01446016
Hydroxychloroquine	lysosomes	no adverse effects observed	HCQ + paclitaxel + carboplatin	advanced or recurrent NSCLC	NCT01649947
		decreased hemoglobin, diarrhea, nausea, vomiting, pain, fatigue, rash	HCQ + capecitabine, oxaliplatin and bevacizumab	metastatic colorectal cancer	NCT01006369
Verteporfin	autophagy formation	no adverse effects observed	verteporfin photodynamic therapy	advanced pancreatic cancer	[149]
Clarithromycin	autophagy flux	anemia, gastrointestinal disorders, dyspnea	clarithromycin + abemaciclib	neoplasm	NCT02117648
		upper respiratory infections, sinus/acute otitis	clarithromycin, dexamethasone, lenalidomide therapy after stem cell transplant	multiple myeloma	NCT00445692
Clarithromycin	autophagy flux	anemia, neutropenia, diarrhea, vomiting, fever, lung infection, renal insufficiency, dehydration, dyspnea	clarithromycin, dexamethasone, pomalidomide	relapsed or refractory myeloma	NCT01159574

**Table 3:** Selected autophagy inhibitor under preclinical investigation.

Autophagy Inhibitor	Chemical Structure	Preclinical Studies	References
3-Methyladenine	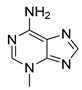	therapy of human ovarian serous papillary cystadenocarcinoma or hepatocellular carcinoma	[155,156]
SAR405	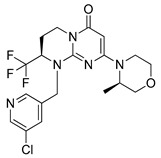	colorectal and melanoma tumors treatment	[159]
Lys05	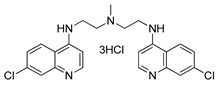	ovarian carcinoma or CML therapy	[160,161,162]
ROC-325	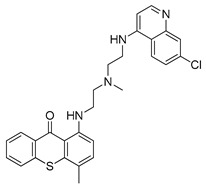	treatment of AML	[164]
Spautin-1	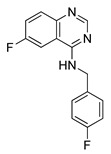	ovarian cancers, CML or prostate cancer treatment	[168,169,170]
MM124	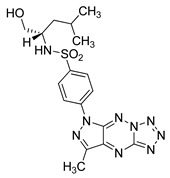	in vitro studies on colon cancer cells	[171]
MM137	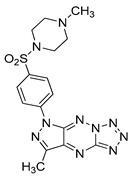	in vitro studies on colon cancer cells	[171]
S130	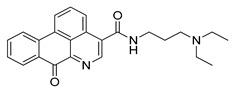	colorectal cancer therapy	[172]
ARN5187	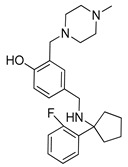	breast cancer treatment	[173,174]
UAMC-2526	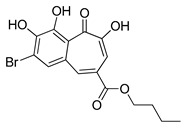	human colon adenocarcinoma treatment	[175]
DCZ5248	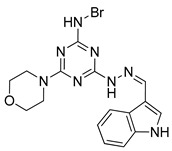	colon cancer therapy	[176]
CA-5f	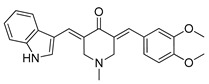	NSCLC therapy	[177]
DQ661	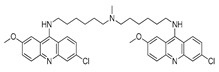	melanoma, colon cancer or pancreatic cancer therapy	[178]
FV-429	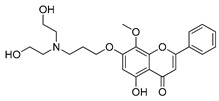	gastric cancers or T-cell malignancies treatment	[179,180]
Madangamine A	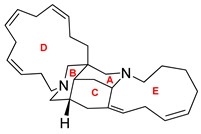	human cervical carcinoma, human fibrosarcoma and human melanoma therapy	[181]

**Table 4:** Selected autophagy activators under clinical investigation.

Autophagy Activator	Chemical Structure	Study Type	References
Rapamycin	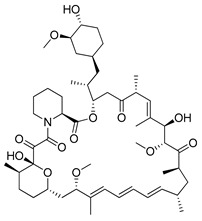	**Clinical trials:** therapy of kaposiform hemangioendothelioma in children, bladder cancer, HER2+ metastatic breast cancer, refractory solid tumors, NSCLC and pediatric relapsed or refractory tumors	NCT04077515, NCT02753309, NCT04375813, NCT04736589, NCT02688881, NCT04348292, NCT02574728
Temsirolimus	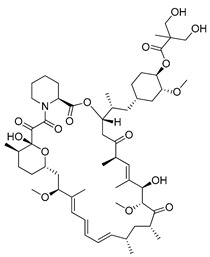	**Preclinical studies:** colorectal cancer, prostate cancer, human papillomavirus-related oropharyngeal squamous cell carcinoma or advanced solid tumors treatment **Clinical trials:** advanced/metastatic malignancies, gynecological malignancies, rare tumors, diffuse intrinsic pontine glioma or solid tumors therapy	[194,195,196,197] NCT01552434, NCT01065662, NCT01396408, NCT02420613, NCT01375829
Everolimus	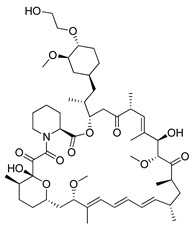	**Preclinical studies:** treatment of advanced papillary variant renal cell carcinoma, triple-negative breast cancer or advanced solid tumors **Clinical trials:** recurrent or progressive ependymoma in children, Hodgkin lymphoma, metastatic transitional cell carcinoma of the urothelium, advanced gynecologic malignancies and breast cancers or recurrent low grade gliomas in young adults and pediatric patients	[199,200,201] NCT02155920, NCT03697408, NCT00805129, NCT03154281, NCT04485559
Metformin	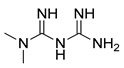	**Clinical trials:** breast cancer, colon cancer, thoracic neoplasm or prostate cancer therapy	NCT04559308, NCT04387630, NCT01980823, NCT04741204, NCT03359681, NCT03477162, NCT02176161, NCT02339168

**Table 5:** Target points, adverse effects and selected therapeutic schemes of autophagy activators.

Autophagy Activator	Target Point	Adverse Effects	Therapeutic Combination or Single-Agent Treatment	Tumor Type	References
Rapamycin	mTOR	blood and lymphatic system disorders e.g., anemia or leukopenia, nausea, fatigue, mucositis, rash	rapamycin + trastuzumab	HER2 receptor positive metastatic breast cancer	NCT00411788
		after treatment with high-dose RAPA (6 mg): neutropenia, diarrhea, fever, stomatitis	rapamycin + radical prostatectomy	advanced localized prostate cancer	NCT00311623
Temsirolimus	mTOR	anemia, abdominal pain, diarrhea, nausea, fever, non-cardiac chest pain, dyspnea, headache, cough, metabolism and nutrition disorders	temsirolimus + sorafenib	thyroid cancer	NCT01025453
		blood and lymphatic system disorders, gastrointestinal disorders, back pain, dizziness, dry skin, pruritus, rash	temsirolimus + bevacizumab	prostate cancer	NCT01083368
Temsirolimus	mTOR	mucositis oral, fatigue, dehydration, dyspnea	temsirolimus + cixutumumab	breast cancer	NCT00699491
Everolimus	mTOR	no adverse effects observed	everolimus and pasireotide	thyroid cancer	NCT01270321
		anemia, vomiting, lower respiratory tract infection, hypercalcemia, confusional state	everolimus + exemestane	estrogen receptor positive advanced breast cancer	NCT01743560
		anemia, abdominal pain, diarrhea, mucositis oral, nausea, vomiting, fatigue, rash	everolimus + pazopanib	solid tumor, kidney cancer	NCT01184326
Metformin	Beclin 1/mTOR	xerostomia, dysphagia, fatigue, dysgeusia	external beam radiation therapy + metformin	head and neck cancer	NCT03109873
		anemia, tinnitus, diarrhea, vomiting, nausea, white blood cell decreased	metformin + cisplatin and radiation therapy	locally advanced head and neck squamous cell carcinoma	NCT02325401

**Table 6:** Selected autophagy activators under preclinical investigation.

Autophagy Activator	Chemical Structure	Preclinical Studies	References
Miconazole	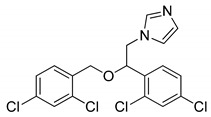	glioblastoma and bladder cancer therapy	[221,222]
CRO15	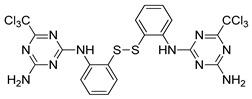	treatment of melanoma	[223]
α-Hederin	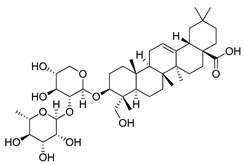	in vitro and in vivo studies on colorectal cancer cells	[226]
MJ-33	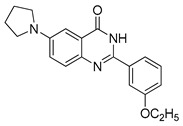	in vitro study on HT-29/5FUR cells	[227,229]
DS00329	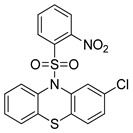	in vitro study on malignant glioblastoma cells	[230]
MHY2256	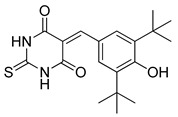	endometrial and colorectal cancer treatment	[231,232]
LCC03	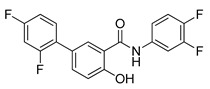	in vitro and in vivo study on castration-resistant prostate cancer	[233]
CZ415	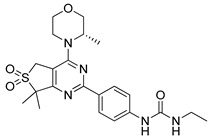	in vitro and in vivo study on human papillary thyroid carcinoma cells	[234]
Eriocalyxin B	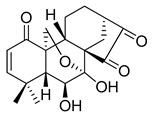	in vitro and in vivo study on breast cancer	[235]

## Data Availability

Not applicable.

## Abbreviations

3-MA	3-Methyladenine
5FUR	5-fluorouracil-resistant
ADCD	autophagy-dependent cell death
AML	acute myeloid leukemia
AMPK	5′ adenosine monophosphate-activated protein kinase
ATG	autophagy-related
AZA	azacitidine
C3G	cyaniding-3-O-glucoside
CAM	clarithromycin
CMA	chaperone-mediated autophagy
CML	chronic myeloid leukemia
CQ	chloroquine
DRAM	damage-regulated autophagy modulator
eMI	endosomal microphagy
ER	endoplasmic reticulum
ESCRT	endosomal sorting complex required for transport
FDA	Food and Drug Administration
HCQ	hydroxychloroquine
Hsc70	heat shock cognate protein 70
JNK-1	C-Jun N-terminal protein kinase 1
LAL	lysosomal acid lipase
LAMP2A	lysosome associated membrane protein type 2A
LC3	light chain protein 3
LDs	lipid droplets
Mcl-1	myeloid cell leukemia 1
MCZ	miconazole
mTOR	mammalian target of rapamycin
NSCLC	non-small cell lung cancer
NUFIP 1	nuclear FMR1 interacting protein 1
PIK3K	phosphoinositide 3-kinase
PMPs	peroxisomal membrane proteins
RAD001	everolimus
RAPA	rapamycin
SQSTM1	sequestosome 1
TEM	temsirolimus
VP	verteporfin
α-HN	α-Hederin
